# Identification and characterization of opportunistic pathogen *Pectobacterium polonicum* causing potato blackleg in China

**DOI:** 10.3389/fpls.2023.1097741

**Published:** 2023-03-03

**Authors:** Wanxin Han, Jinhui Wang, Minna Pirhonen, Yang Pan, Jingxin Qin, Shangqing Zhang, Jiehua Zhu, Zhihui Yang

**Affiliations:** ^1^College of Plant Protection, Hebei Agricultural University, Baoding, China; ^2^Department of Agricultural Sciences, University of Helsinki, Helsinki, Finland; ^3^Institute of Plant Protection, Tangshan Academy of Agricultural Sciences, Tangshan, China

**Keywords:** *Pectobacterium*, potato, blackleg, virulence, MLSA, WGS, genome comparison

## Abstract

Blackleg and aerial stem rot of potato (*Solanum tuberosum* L.), caused by soft rot enterobacteria of the genera *Pectobacterium* and *Dickeya*, has recently increased years in Hebei Province, China. Field surveys were performed during the 2021 potato growing season in Hebei to identify and characterize bacterial pathogens. Sixteen potato plants showing blackleg or aerial stem rot were collected from three potato-producing areas, and ten representative pectinolytic bacteria were isolated from symptomatic plants. 16S rDNA sequencing and multilocus sequence analysis were performed to determine the taxonomic position of the bacterial isolates. The isolates belonged to the genus *Pectobacterium*, including *Pectobacterium atrosepticum*, *Pectobacterium carotovorum*, *Pectobacterium brasiliense*, and *Pectobacterium parmentieri*. The exceptions were isolates BY21311 and BY21312, which belonged to a new species of *Pectobacterium polonicum* previously found in groundwater. The taxonomy of isolate BY21311 was confirmed using whole genome-based analysis. *P. polonicum* has only been identified in potato plants on one farm in Baoding region in China. Isolates BY21311 and BY21312 displayed similar physiological and biochemical traits to the type strain DPMP315^T^. Artificial inoculation assays revealed that isolate BY21311 fulfilled Koch’s postulates for potato blackleg. These findings represent the first time *P. polonicum*, a water-associated *Pectobacterium* species may be the cause of blackleg in the field. Interestingly, *P. polonicum* BY21311 has reduced ability to macerate potato tubers when compared to *P. atrosepticum*, *P. brasiliense*, *P. versatile*, and *P. parvum*, which is more virulent in tubers than the type strain DPMP315^T^. The host range of isolate BY21311 was determined by injection method, which can impregnate five plants. Although the genome of isolate BY21311 harbors gene clusters encoding a type III secretion system, it did not elicit a hypersensitive response (HR) in *Nicotiana benthamiana* or *N. tabacum* leaves. T3SS effector AvrE and T4SS effector PilN were obtained by predicting isolate BY21311 genome. *P. polonicum* appears to show significant variations in gene content between two genomes, and gene content varies between isolates BY21311 and DPMP315^T^, with strain specific-genes involved in many aspects, including lipopolysaccharide biosynthesis, substrate translocation, T4SS and T6SS among others, suggesting that isolates BY21311 and DPMP315^T^ might represent distinct clades within the species.

## Introduction

The genera *Pectobacterium* and *Dickeya*, also referred to as soft rot *Pectobacteriaceae* (SRP), are the primary pathogens causing soft rot diseases in several plant hosts (Ma et al., 2007; Charkowski, 2018). SRP causes blackleg and aerial stem rot in the field and tuber soft rot in the field and storage, leading to substantial economic losses in potato production worldwide. Unlike the situation in Europe and the United States, where *Dickeya* species has emerged as a significant threat to potato production (Toth et al., 2011; Ma et al., 2018; Curland et al., 2021), the causal pathogens of potato soft rot diseases reported in China have all been *Pectobacterium* species to date. Although *Dickeya* species have been reported in China to cause of soft rot in rice (Pu et al., 2012), banana (Zhang et al., 2014), pear (Tian et al., 2016), ornamental plants (Lin et al., 2012; Zhou et al., 2012) and sweet potato (Huang et al., 2010), *Dickeya* has not yet been identified in potato plants. *P. atrosepticum* and *P. carotovorum* were once regarded as the most frequently isolated pathogens that are associated with potato soft rot diseases in China (Zhang et al., 2012; She et al., 2013). In recent years, other *Pectobacterium* species, including *P. brasiliense* (Zhao et al., 2018; Jiang et al., 2019), *P. parmentieri* (Cao et al., 2021), *P. versatile* (Han et al., 2022), *P. polaris* (Handique et al., 2022a) and *P. punjabense* (Handique et al., 2022b), have also been reported in the primary potato growing areas in China. Two citrate negative isolates of *P. polaris* were reported to cause aerial stem rot in Hebei Province, and subsequent genome-based analysis confirmed that they belonged to the newly established species, *P. parvum* (Wang et al., 2022a; Wang et al., 2022b).

The *Pectobacterium* genus has been enlarged with several new species over the past few years. As of the end of 2019, there were 18 validly published species were in the genus (Charkowski, 2018; Sarfraz et al., 2020). In 2020, an atypical group of *P. polaris*, was elevated to the species level as *P. parvum* sp. nov (Pasanen et al., 2020). In 2021, *P. quasiaquaticum* sp. nov. was established, representing a group of strains isolated from waterways in France (Moussa et al., 2021). Within the genus, *P. quasiaquaticum* is closely related to *P. aquaticum*, which also has a freshwater origin (Pedron et al., 2019; Babinska et al., 2021). To date, a total of 11 *Pectobacterium* species have been reported in potato plants, including *P. aroidearum*, *P. atrosepticum*, *P. betavasculorum*, *P. brasiliense*, *P. carotovorum*, *P. parmentieri*, *P. peruviense*, *P. parvum*, *P. polaris*, *P. punjabense*, and *P. versatile* (Pasanen, 2020; Pasanen et al., 2020).

DNA sequence-based methods are used to detect and characterize of bacteria. The 16s rDNA universal primers (Weisburg et al., 1991) are the most widely used for rapid diagnosis; however, the 16S rDNA has poor discriminatory power and may not be reliable at the species or subspecies level (Drancourt et al., 2000; Mignard and Flandrois, 2006; Naum et al., 2008). Therefore, PCR assays based on other housekeeping genes have been developed for more specific detection of *Pectobacterium* species, for example, *pel* (Darrasse et al., 1994), *recA* (Waleron et al., 2002), *pmrA* (Kettani-Halabi et al., 2013), *gapA* (Cigna et al., 2017), and *dnaA* (Dobhal et al., 2020). Multilocus sequence analysis (MLSA) schemes have also been established (Ma et al., 2007; Waleron et al., 2008; Nabhan et al., 2012; Waleron et al., 2018; Oulghazi et al., 2019) because concatenation or coalescence of multiple gene loci yields more accurate phylogenetic inference. The implementation of whole genome sequencing (WGS) in SRP has fundamentally improved the phylogenetic inference of *Pectobacterium* species, and WGS of bacteria has now become the new gold standard for species delineation (Zhang et al., 2016; Portier et al., 2020; Jonkheer et al., 2021).

The incidence of blackleg and aerial stem rot of potatoes has increased over recent years in northern China, and SRP has caused significant yield loss in highly susceptible cultivars, including Xisen 6, Huangxin 226, Helan 15 (also known as Favorita), and others (Zhao et al., 2018; Cao et al., 2021; Han et al., 2022; Handique et al., 2022a; Wang et al., 2022a; Handique et al., 2022b). In 2021, potato blackleg and aerial stem rot were reported in the three primary production areas in Hebei Province. Diseased plants were sampled during field surveys, and *Pectobacterium* species were isolated from plant tissue and subsequently identified using 16S rDNA sequencing and MLSA. In the present study, *P. polonicum*, a new *Pectobacterium* species discovered from groundwater in Poland (Waleron et al., 2019), was identified by MLSA and further confirmed using WGS and genome-based analysis. Koch’s postulates were fulfilled, confirming *P. polonicum* as an opportunistic pathogen cause of potato blackleg. Physiological characteristics and virulence traits of the species were also studied. We also compared the genomes of *P. polonicum* isolates DPMP315^T^ and BY21311 to reveal gene content differences.

## Materials and methods

### Plant samples and bacterial isolates

In 2021, symptomatic potato plants were collected from three regions in Hebei Province (**Supplementary Table S1**). Potato plants showing blackleg were observed in a village in Yutian County, Tangshan region. The disease incidence in that field was approximately 25%, and the size of the field size was 3 ha. The cultivar was Helan 15 (Favorita), and we collected five potato plants there. Chinese cabbage (Brassica rapa subsp. pekinensis) was grown in the neighboring fields. Potato blackleg was also found in an organic farm in Boye County, Baoding region. The size of the potato field was 2 ha, two cultivars (cv. Xisen 6 and Helan 15) were grown, and the disease incidence was approximately 40%. We collected six potato plants there. Common vegetables, tomato (*Solanum lycopersicum*), cucumber (*Cucumis sativus*), celery (*Apium graveolens*), garlic (*Allium sativum*), and others were grown in greenhouses which were located adjacent to the potato field. Potato plants showing aerial stem rot were sampled from greenhouses in a farm in Zhangbei County, Zhangjiakou region. Ten greenhouses grew potatoes (cv. Shepody) on that farm. The size of each greenhouse was 0.03 ha, and aerial stem rot was found in two greenhouses with disease incidence over 30%. We collected five potato plants there. All affected stem tissues were surface sterilized by dipping them in 75% ethanol for 1 min followed by three successive rinses with sterile distilled water. Then, the stem tissues were cut into pieces of approximately 0.5 cm in length and were soaked in 0.9% NaCl for 20 min. Ten-fold dilutions of the tissue specimen soaking solution (20 µl) were plated onto semi-selective crystal violet pectate (CVP) agar medium (Hélias et al., 2012), and plates were incubated at 28°C for 48 h. Bacterial colonies producing pits on CVP were restreaked and purified on Luria-Bertani (LB) agar plates, and single colonies were restreaked to fifth generations.

### 16S rDNA sequence analysis

The pectinolytic isolates were first identified using 16S rDNA sequencing. The bacterial gDNA was isolated using an Easy-Pure Bacteria Genomic DNA Kit (TransGen Biotech) according to the protocol provided by the manufacturer. The 16S rDNA region was amplified by conventional PCR using the universal primers 27F and 1492R (Weisburg et al., 1991). The PCR reaction was performed using Taq Plus Master Mix (Vazyme Biotech). The PCR program was 94°C for 5 min, 30 cycles of 94°C for 30 s, 58°C for 30 s, 72°C for 50 s, and 72°C for 10 min. The PCR products were purified using a SanPrep Column DNA Gel Extraction Kit (Sangon Biotech). The purified PCR amplicons were sequenced in the forward and reverse directions (Sangon Biotech). The sequenced reads were assembled into consensus sequences with primer trimming using Contig Express from the Vector NTI Suite v6.0. The 16S rDNA sequence for each isolate was searched against the NCBI 16S rRNA database using BLASTn.

### MLSA

To assign species-level taxonomy, a MLSA of the concatenation of six housekeeping genes (*acnA*, *gapA*, *icdA*, *mdh*, *proA*, and *rpoS*) of the isolates (**Supplementary Table S5**) (Ma et al., 2007; Waleron et al., 2008) was performed in comparison with the type strains of *Pectobacterium* species (**Supplementary Table S2**). The genome assemblies available from NCBI RefSeq (as of April 2022) were used for MLSA and genome-based analysis. The PCR program was as follows: 94°C for 5 min, 35 cycles of 94°C for 30 s, 55°C for 30 s, 72°C for 50 s, and 72°C for 10 min. The PCR products were purified and sequenced as described above. Each individual MLSA locus was aligned with previously sequenced *Pectobacterium* and *Dickeya* species (**Supplementary Table S2**) using MAFFT v7.490. To avoid codon-breaking nucleotide alignments caused by indels, the MLSA nucleotide sequences were first translated into amino acid sequences, and these sequences were aligned in MAFFT; then, the amino acid alignments were back-translated to corresponding nucleotide alignments. Finally, the alignments of six loci were concatenated to form a supermatrix using FASconCAT v1.11. If the nucleotide sequence of the concatenated loci from different isolates were identical, then the isolates were merged as one operational taxonomic unit in the phylogenetic tree. To select the best-fit nucleotide substitution model for the supermatrix, model selection was performed using jModelTest v2.1.10. A maximum-likelihood tree was constructed using RAxML v8.2.12 and applying the ‘GTRGAMMAI’ setting. To estimate the sequence similarity between the isolate BY21311 and *P. polonicum*, and between BY21311 and *P. punjabense*, the MLSA sequences of isolate BY21311 were compared with *P. polonicum* and *P. punjabense* type strains using BLASTn.

### WGS and genome-based analysis

WGS was performed to resolve the phylogenetic position of isolate BY21311, and to reveal intraspecific variations of *P. polonicum*. Because the concatenated sequence of isolate BY21311 is identical to that of isolate BY21312, thus, isolate BY21311 was selected and sequenced as a representative strain. For gDNA extraction, isolate BY21311 was grown in LB liquid medium with shaking (200 rpm) at 28°C until it reached an OD_600_ of 0.7. Bacterial DNA was extracted using an Easy-Pure Bacteria Genomic DNA Kit (TransGen Biotech). The bacterial genome was sequenced using a hybrid approach using Oxford Nanopore PromethION 48 and Illumina NovaSeq 6000. Two sequencing libraries were constructed using SQK-LSK109 and EXP-NBD104/114 Kits for PromethION and KAPA HyperPlus Kit for NovaSeq, respectively. The library insert size for Illumina paired-end (150 bp) sequencing was 400 bp. *De novo* assembly of Nanopore long reads was performed using Canu v2.2. Illumina reads were adapter-clipped and quality-trimmed using fastp v0.23.2. The genome assembly was polished with Illumina short reads using Pilon v1.24 to improve accuracy, and no further error correction was observed after four rounds of polishing. The completeness of the final assembly was evaluated using BUSCO v5.2.2 (Manni et al., 2021), and the genome sequence was annotated using the NCBI Prokaryotic Genome Annotation Pipeline (PGAP). The core gene set of *Pectobacterium* species (**Supplementary Table S2**) for phylogenetic inference was determined by mapping to the 400 universal markers (Segata et al., 2013; Zhu et al., 2019) available in PhyloPhlAn v3.0(Asnicar et al., 2020). A phylogenic tree for *Pectobacterium* species was constructed, using the supermatrix approach and based on 376 universal markers. The supermatrix’s best amino acid substitute model for the supermatrix was determined using ProtTest v3.4.2. The average nucleotide identity (ANI) values between species were calculated with pyani v0.2.11 using the ANIm algorithm (Pritchard et al., 2016). *In silico* DNA-DNA hybridization (*is*DDH) (Meier-Kolthoff et al., 2013) was calculated with the GGDC v3.0 using the BLAST+ alignment. To determine the correlation pattern between species and strains based on their ANI and DDH values, heat maps were drawn. Isolate BY21311 genome using the T3SEpp (Hui et al., 2020) and Eff3ctidor (Wagner et al., 2022) to predict Type III effectors, and using T4SEfinder (Zhang et al., 2022) to predict Type IV effectors. To better understand the differences in gene content between *P. polonicum* BY21311 and DPMP315^T^, genomic comparison of two stains analysis was performed using the Prokka-Roary pipeline (Seemann, 2014; Page et al., 2015). BY21311 and DPMP315^T^ genomes were annotated using Prokka v1.14.6, and ortholog groups were determined using Roary v3.13.0.

### Physiological and biochemical phenotypes

The physiological and biochemical traits of isolates BY21311 and BY21312 were determined using Biolog GEN III MicroPlate (Biolog, Hayward, CA). Isolates were streaked twice on Biolog Universal Growth (BUG) agar, and colonies were picked and added to a fresh IF-A glass tube to adjust the bacterial density to 98%T with Biolog Turbidimeter. The prepared bacterial suspension (100 µl/well) was added to GEN III 96-well plate. After incubation at 30°C for 24 hours, color formation in each well was measured using a Biolog OmniLog reader. The physiological and biochemical phenotypes of the novel species were compared to the closely-related species: *P. polonicum*, *P. punjabense*, *P. parmentieri* and *P. wasabiae* (Sarfraz et al., 2018; Waleron et al., 2019; Cigna et al., 2021).

### Artificial inoculation of potato stems and tubers

To demonstrate the pathogenicity of *P. polonicum* isolate BY21311 in potato plants, potato seedlings (cv. Xisen 6) were grown in a greenhouse for five weeks and subsequently inoculated with isolate BY21311. The isolate was grown in LB liquid medium with shaking (200 rpm) at 28°C for 6 hours. Bacterial cells were collected by centrifugation (9000 rpm for 1 min). The cells were washed once with 10 mM MgSO_4_ buffer and resuspended in the same buffer and adjusted to an OD_600_ of 0.80. In a potato plot in a greenhouse, six potato plants were randomly selected. The bacteria suspension (100 µl) was injected into the aboveground stem base of three potato plants, and the MgSO_4_ buffer was injected into the other three potato plants as a negative control. Seal with vaseline immediately after injection. Plants were grown in a greenhouse at 30°C with 12/12 h light-dark cycles for two weeks. DNA was extracted from diseased tissues of diseased tissue. The extracted DNA were identified using *rpoS* and *gapA* primers.

A virulence assay was performed as described to evaluate the maceration ability of isolate BY21311 on potato tubers, virulence assay was performed as described (Nykyri et al., 2013; Pasanen et al., 2020). Isolate BY21311 was compared with isolates of *P. atrosepticum* (the strain isolated by our lab in 2020 was used as a positive reference), *P. versatile* (Han et al., 2022), *P. brasiliense* and *P. parvum* (Wang et al., 2022a) which were tested in 2020. Potato tubers (cv. Xisen 6) were washed with tap water, surface sterilized with 0.6% NaClO for 7 min, and washed three times with sterile distilled water, and air dried on a clean bench. Bacterial isolates were grown in LB liquid medium with shaking (200 rpm) at 28°C overnight. Bacterial cells were collected by centrifugation, and the cells were washed once with the MgSO_4_ buffer, resuspended in the same buffer, and adjusted to an OD_600_ of 0.27. Potato tubers were stabbed with a pipette tip (200 µl) to create a cavity, and 50 µl of bacterial suspension was inoculated. A group of potato tubers was inoculated with the MgSO_4_ buffer as a buffer control. The wounds were sealed with Vaseline (Qingdao Hainuo). The inoculated tubers were wrapped with wet kitchen paper, placed into plastic containers, and sealed with tape. The boxes were placed in the dark at room temperature (23-25 °C) for 72 hours. After incubation, the potato tubers were cut in half, and the rotten tissue was scraped off with a spoon and weighed. The mean weight of tissue debris scraped from the buffer control group was used as a baseline to adjust the weight of macerated tissue of other treatments. One-way analysis of variance (ANOVA) followed by *post-hoc* tests was performed using R v4.1.2 to determine whether there were significant differences in virulence exist among isolates. Similar results were obtained from three independent experiments.

### Hypersensitive response (HR) in nonhost plants

To determine whether isolate BY21311 can elicit a HR in non-host tobacco, *Nicotiana benthamiana* and *N. tabacum* plants were grown in a growth chamber at 25°C with 12/12 h light-dark cycles for 4-5 weeks. It is known that *P. brasiliense* can elicit HR in *N. benthamiana* and *N. tabacum* leaves (Kim et al., 2009; Maphosa and Moleleki, 2021) but *P. parvum* does not (Pasanen et al., 2020); therefore, *P. brasiliense* isolate FR19412 and *P. parvum* isolate FN20211 were used as positive and negative controls, respectively. Bacterial isolates were grown overnight at 28°C in LB liquid medium, and bacterial cells were washed once with 10 mM MgSO_4_ buffer, resuspended in the same buffer and adjusted an OD600 of 0.14. N*. benthamiana* and *N. tabacum* leaves were infiltrated with bacterial suspensions using a syringe without a needle, and the same MgSO_4_ buffer was used as a buffer control. The inoculated tobaccos were grown in a growth chamber at 25°C with 12/12 h light-dark cycles for 72 hours.

### Host range determination

To determine the host range of *P. polonicum* isolate BY21311, baby bok choy (*Brassica campestris* L. ssp. *chinensis* Makino var. *communis* Tsen et Lee), baby Chinese cabbage, *Brassica chinensis* L., and *Cucumis sativus* L. are planted and grown in a greenhouse for 7 weeks, plus vitro tomatoes (*Solanum lycopersicum* L.) and subsequently inoculated isolate BY21311. The isolate was grown in LB liquid medium with shaking (200 rpm) at 28°C for 6 hours. Bacterial cells were collected by centrifugation (9000 rpm for 1 min). The cells were washed once with 0.9% NaCl buffer and resuspended in the same buffer and adjusted to an OD600 of 0.80. The bacteria suspension (100 µl) was injected into the stem or fruit of vegetables, and the 0.9% NaCl buffer as a negative control. Seal with Vaseline immediately after injection. Tomatoes were grown in a greenhouse at 20-25°C with 12/12 h light-dark cycles for 3 days and other vegetables were grown for 7 days.

## Results

### Identification of *Pectobacterium* isolates

Ten pectinolytic isolates were obtained after five rounds of single-colony purification. The BLASTn results of the 16S rDNA amplicons of the pectinolytic isolates revealed that they all belonged to the genus *Pectobacterium* (**Supplementary Table S1**). Four pectinolytic isolates (YT21111, YT21121, YT21211, and YT21222) were isolated from five potato plants in Tangshan. A total of four pectinolytic isolates (BY21121, BY21221, BY21311, and BY21312) were isolated from six potato plants in Baoding. Two pectinolytic isolates (ZB21311 and ZB21312) were isolated from two potato plants in Zhangbei, which were two strains *P. brasiliense*.

The result revealed that MLSA, based on six housekeeping genes (*acnA*, *gapA*, *icdA*, *mdh*, *proA*, and *rpoS*), assigned the isolates to five known *Pectobacterium* species (**Supplementary Table S1**; **Supplementary Figure 1**). Two isolates belonged to *P. atrosepticum* (YT21111 and YT21121), two to *P. brasiliense* (ZB21311 and ZB21312), two to *P. polonicum* (BY21311 and BY21312), two to *P. carotovorum* (BY21121 and BY21221), and two to *P. parmentieri* (YT21211 and YT21222). An interesting finding in this isolate collection was *P. polonicum*, identified initially from groundwater sampled from a vegetable field in the Northern Poland (Waleron et al., 2019). The MLSA sequences of isolate BY21311 were more similar to *P. polonicum* type strain DPMP315^T^ (The average “Query Cover” and “Sequence Identity” of six loci were 100% and 99.87%, respectively) than to *P. punjabense* type strain SS95^T^ (The average “Query Cover” and “Sequence Identity” of six loci were 99% and 97.65%, respectively) (**Table 1**). The maximum-likelihood method was used to reconstruct the phylogenetic tree (**Supplementary Figure 1**), revealing that *P. punjabense* and *P. polonicum* are closely related sister species.

**Table 1:** BLASTn results of MLSA sequences of *Pectobacterium* isolate BY21311 to *P. polonicum* and *P. punjabense*.

Strain^a^	Isolation Source	Geographical Origin, Year of Isolation	Gene Locus	Accession Number (Locus tag)	Query Coverage	Sequence Identity	Reference
*P. polonicum* BY21311	potato	China 2021	*rpoS*	OM044551 (765bp)	–	–	–
			*proA*	OM044552 (699bp)	–	–	
			*gapA*	OM044553 (495bp)	–	–	
			*icdA*	OM044556 (546bp)	–	–	
			*acnA*	OM044555 (354bp)	–	–	
			*mdh*	OM044554 (510bp)	–	–	
*P. punjabense* SS95^T^	potato	Pakistan 2017	*rpoS*	E2566_16880	100%	97.65%	Sarfraz et al., 2018
			*proA*	E2566_16520	100%	91.56%	
			*gapA*	E2566_10485	99%	98.10%	
			*icdA*	E2566_10015	100%	94.41%	
			*acnA*	E2566_12300	98%	93.02%	
			*mdh*	E2566_03350	100%	91.46%	
*P. polonicum* DPMP315^T^	groundwater in vegetable field	Poland 2016	*rpoS*	EDI29_13735	100%	99.87%	Waleron et al., 2019
			*proA*	EDI29_17855	100%	98.98%	
			*gapA*	EDI29_02940	99%	99.97%	
			*icdA*	EDI29_02475	100%	99.44%	
			*acnA*	EDI29_04400	100%	99.72%	
			*mdh*	EDI29_11450	100%	100%	

a The superscript ‘T’ indicates type strain.

### Genome sequence and *in silico* analyses

**Figure 1:**
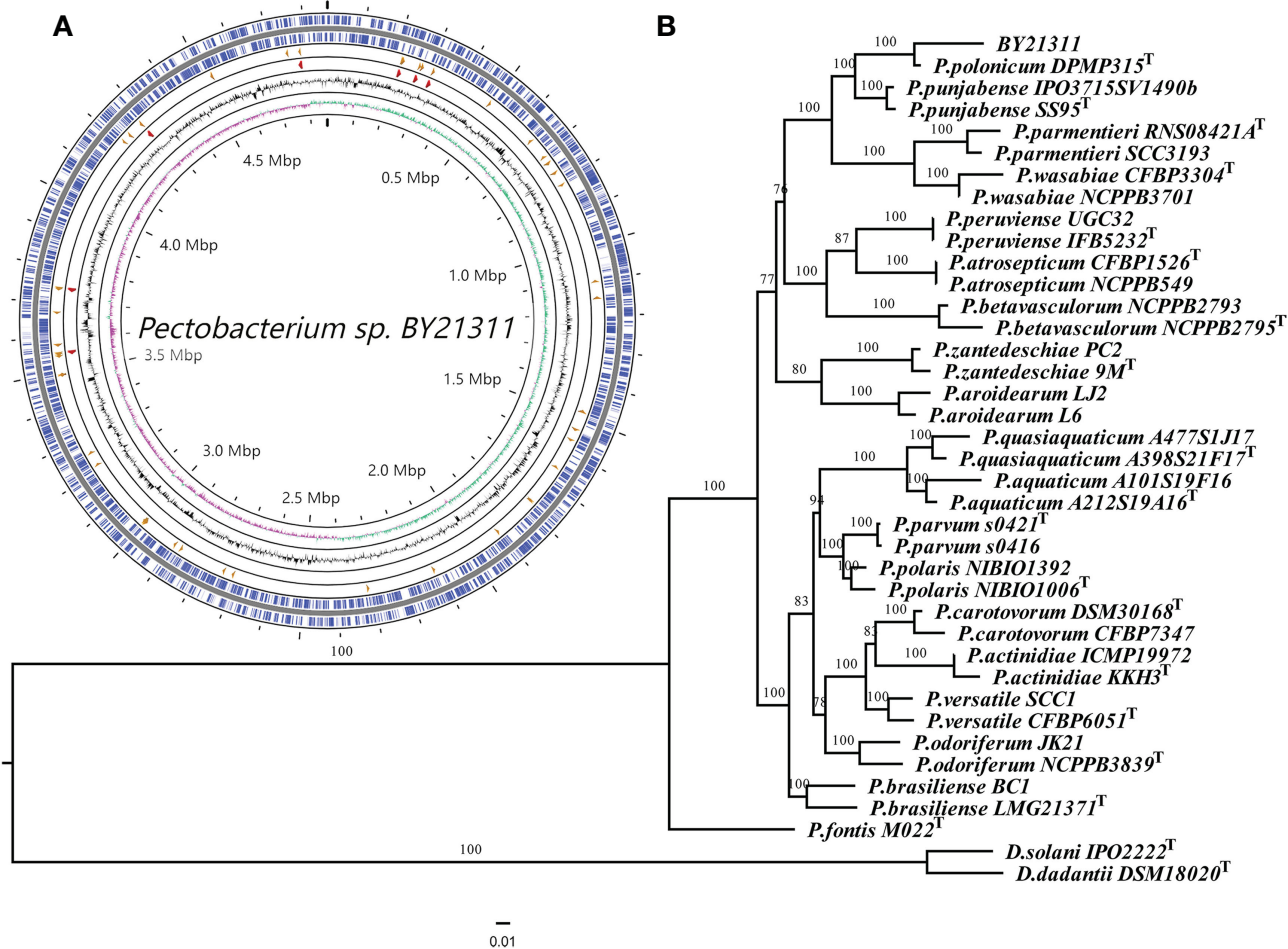
Genome map of *Pectobacterium* isolate BY21311 and genome-based phylogenetic inference. **(A)** Circular representation of the complete genome assembly of isolate BY21311. The circles represent (from inner to outer) GC skew, GC%, rRNAs, tRNAs, and protein-coding genes on the forward and reverse strands. **(B)** Maximum likelihood (ML) tree showing the phylogenetic relatedness between isolate BY21311 and other closely related *Pectobacterium* species, based on the concatenated sequences of 376 conserved genes. *Dickeya solani* and *D. dadantii* were selected as outgroup. ML tree was constructed using RAxML v8.2.12 with ‘PROTGAMMAJTTF’ setting. The superscript ‘T’ in the tree indicates type strain. The branch lengths indicate the evolutionary distance as the number of amino acid substitutions per site. The numbers shown next to the branches indicate the percentage of bootstrap support values (1000 replicates).

Illumina NovaSeq generated 22,938,662 paired-end short reads, and Nanopore produced 115,854 long reads. The coverage of Nanopore long reads and Illumina short reads to the final genome assembly of isolate BY21311 were 224x and 617x, respectively. The circular chromosome of isolate BY21311 (CP090065.1) was 4,863,665 bp in length, which is slightly longer than the *P. polonicum* type strain DPMP315^T^ (4,836,128 bp); however, the GC content of isolate BY21311 (51.08%) was slightly lower than that of DPMP315^T^ (51.28%) (**Supplementary Table S3**; **Figure 1A**). No plasmid was assembled from the sequencing data. The complete genome of isolate BY21311 harbors 4,178 protein-coding genes, 22 genes encoding rRNAs and 77 genes encoding tRNAs (**Supplementary Table S3**).

**Figure 2:**
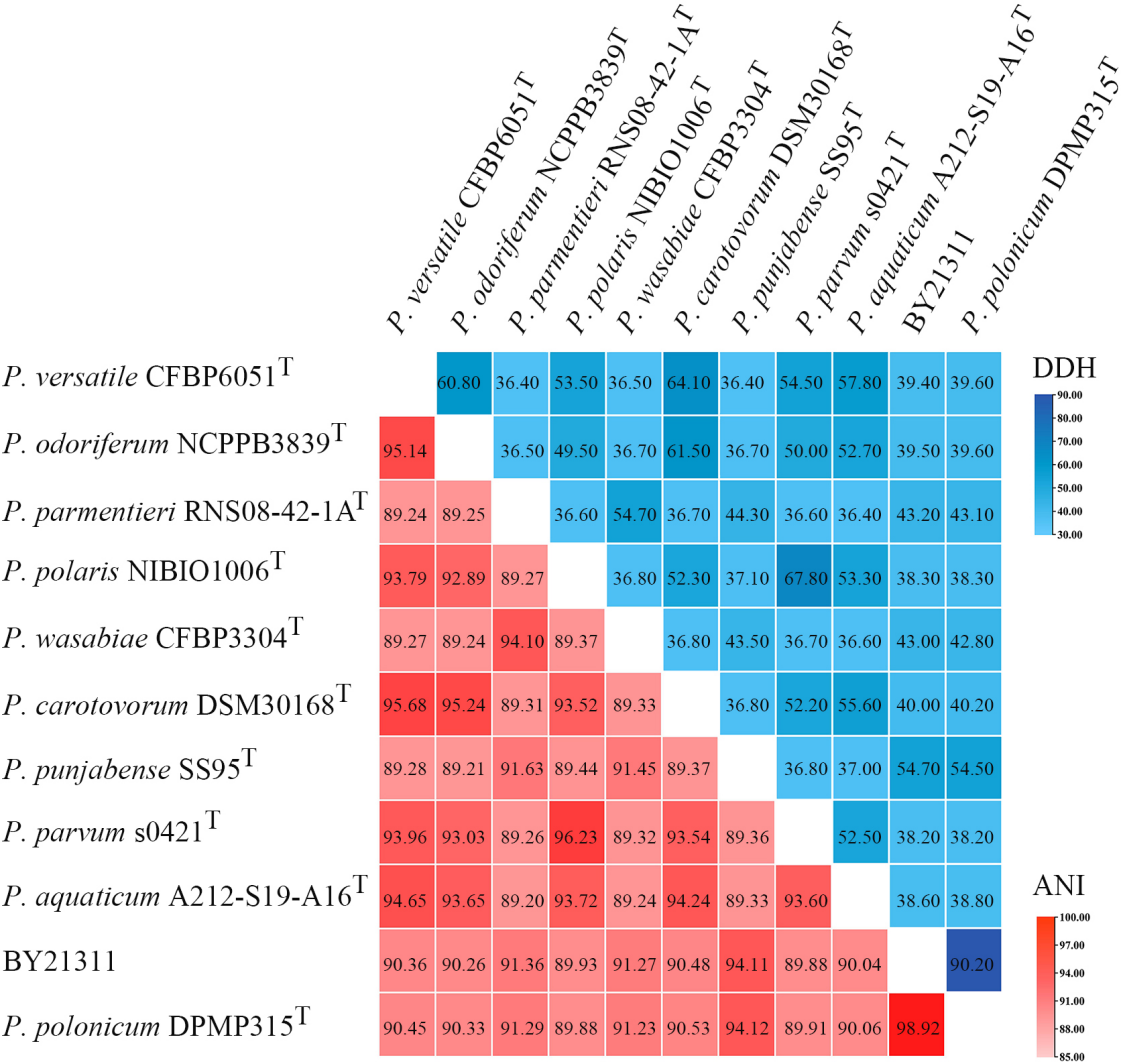
Heat map of average nucleotide identity (ANI) values and DNA-DNA hybridization (DDH) values compared among 11 related strains. ANI and DDH values are indicated by the color intensity.

Based on 376 universal genes, the phylogenetic tree clearly shows that isolate BY21311 and *P. polonicum* DPMP315^T^ were clustered together and formed a sister clade to *P. punjabense* strains with strong bootstrap support (**Figure 1B**). ANI and *is*DDH values (**Figure 2**) of isolate BY21311 to its closest *Pectobacterium* species, *P. polonicum* DPMP315^T^ was 98.92% and 90.20%, respectively, which are above the empirical cut-off (ANI > 95-96% and *is*DDH > 70%) for species delineation (Richter and Rosselló-Móra, 2009; Auch et al., 2010). These findings suggest that isolate BY21311 belongs to *P. polonicum*.

The bacterial effectors predicted by T3SEpp and Eff3ctidor were the AvrE (Locus_tag: LW347_RS11220). One type IV effector PilN (Locus_tag: LW347_RS04650) were predicted by T4SEfinder.The genomes of *P. polonicum* DPMP315^T^ and BY21311, representing strains isolated from groundwater and potato stem, respectively, share a core genome consisting of 3,917 ortholog groups, and forming a genomic comparison of two stains that consisting of 4,634 ortholog groups (**Supplementary Table S4**). There were 392 and 325 protein-coding genes that were present only in DPMP315^T^ or BY21311, which constitute 8.46% and 7.01%, respectively, of the genomic comparison of two stains. Currently, only two genome sequences of *P. polonicum* are available; however, *P. polonicum* tends to have significant variations in gene content between two genomes because the fact that the fraction of strain-specific genes is relatively large. Most of the strain-specific genes (429/717) were annotated as hypothetical proteins with unknown functions. In addition, 50 out of 717 genes were transposons, phages or prophage-related, and most of them (40/50) were present in DPMP315^T^ but not in BY21311, suggesting that DPMP315^T^ genome had undergone extensive recombination, insertion, and gene transfer. DPMP315^T^ and BY21311 varied in genes (30/717) encoding transporters that may be involved in the translocation of different substrates, genes encoding various types of transcriptional regulators (19/717), and bacterial toxin-antitoxin system (18/717). Interestingly, these two isolates also varied in lipopolysaccharide (LPS) and LPS O-antigen biosynthesis, part of bacterial outer membrane biogenesis; BY21311 harbors extra seven genes involved in LPS biosynthesis. DPMP315^T^ harbors one extra gene encoding the Type VI secretion system (T6SS) protein VgrG and two extra T6SS effectors; by comparison, BY21311 harbors three extra genes encoding T4SS proteins.

### Physiological and biochemical phenotypes

Two isolates (BY21311 and BY21312) showed the same reaction pattern in Biolog GEN III MicroPlate. They exhibited almost identical physiological and biochemical traits to *P. polonicum* DPMP315^T^ based on the following items (**Table 2**): negativity for utilization of inosine, sorbitol, maltose and 3-methyl-glucose; and positivity for utilization of raffinose, melibiose, lactose, galactose, mannose, cellobiose, and citric acid. *P. polonicum* DPMP315^T^ was negative for utilization of glucuronamide and sensitive to LiCl, which are different from *P. punjabense* SS95^T^; however, the same results were not observed in isolates BY21311 and BY21312.

**Table 2:** Physiological and biochemical characteristics of *Pectobacterium* species.

Test	Bacterial strain^a^					
	*P. polonicum* BY21311	*P. polonicum* BY21312	*P. polonicum* DPMP315 ^T^	*P. punjabense* SS95^T^	*P. parmentieri* RNS 08-42-1A^T^	*P. wasabiae* CFBP 3304^T^
Inosine	–	–	–	–	–	–
D-raffinose	+	+	+	+	+	–
D-melibiose	+	+	+	+	+	–
α-D-lactose	+	+	+	+	+	–
D-galactose	+	+	+	+	+	+
D-sorbitol	–	–	–	–	–	–
D-maltose	–	–	–	–	–	–
D-mannose	+	+	+	+	+	+
D-cellobiose	+	+	+	+	+	+
3-Methyl-glucose	–	–	–	–	–	–
L-alanine	+/-	–	–	–	–	–
Citric acid	+	+	+	+	+	–
Glucuronamide	+/-	+/-	–	+	–	+
Lithium chloride	+/-	+/-	–	+	–	+

a The corresponding phenotypes of *P. polonicum* DPMP315^T^, *P. punjabense* SS95^T^, *P. parmentieri* RNS 08-42-1A^T^ and *P. wasabiae* CFBP 3304^T^ were summarized from Sarfraz et al. (2018), Waleron et al. (2019) and Cigna et al. (2021). +, positive; −, negative; +/−, borderline.

### Artificial inoculation of potato stems and tubers

**Figure 3:**
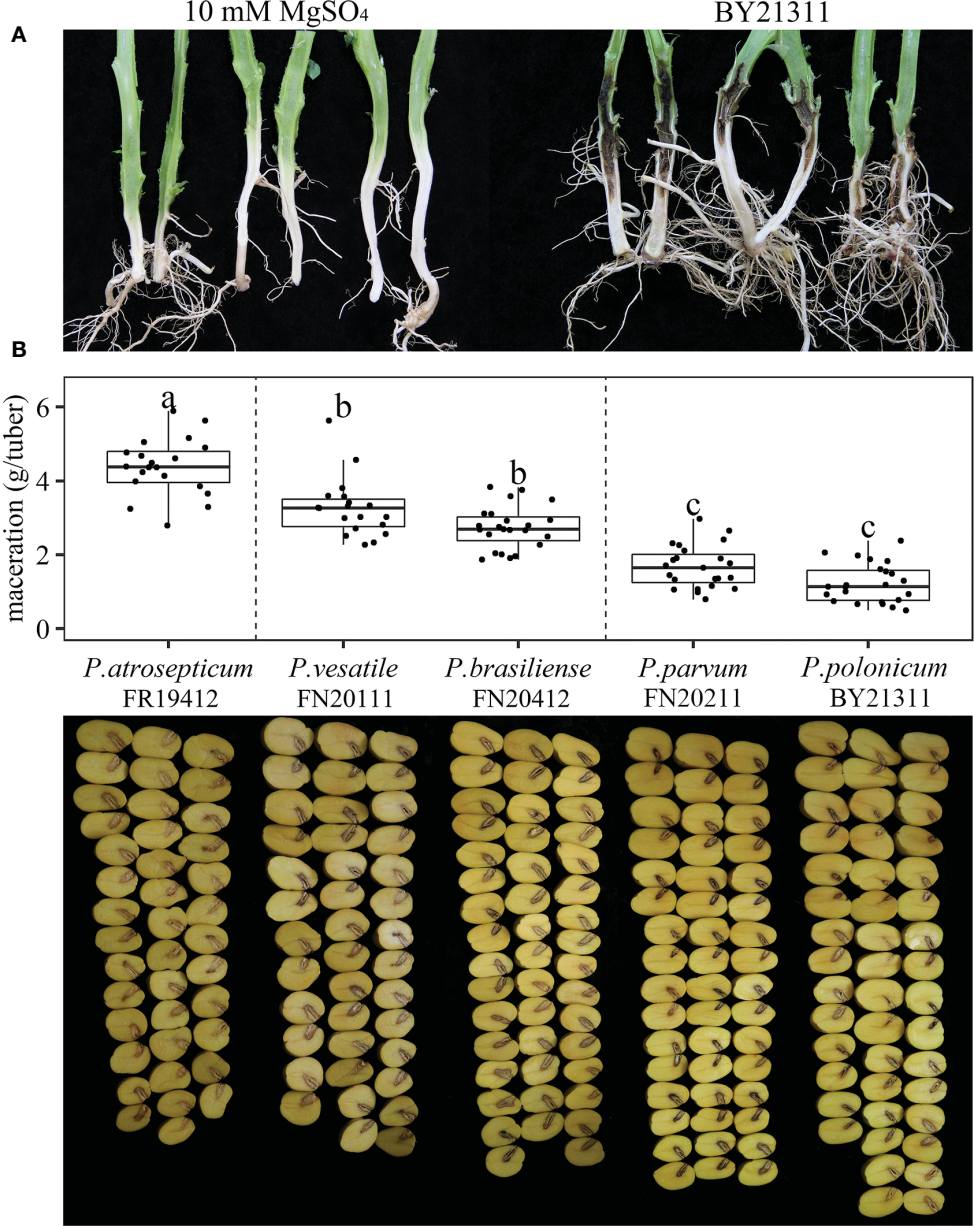
Pathogenicity test of *Pectobacterium polonicum* isolate BY21311 on potato stems and maceration assays of *Pectobacterium* species on potato tubers. **(A)** Potato stems were inoculated with isolate BY21311, and 10 mM MgSO4 solution was used as buffer control. Stems were cut open at two weeks after inoculation. **(B)** Washed and surface sterilized potato tubers were inoculated with *Pectobacterium* species. Tubers were cut open at 72 hours after inoculation. Boxplot showing variations in maceration ability on potato tubers among *P. atrosepticum* isolate FN20412, *P. versatile* isolate FN20111, *P. brasiliense* isolate FR19412, *P. parvum* isolate FN20211 and *P. polonicum* isolate BY21311. The black dots in each category (box) represent the observations. One-way analysis of variance (*F* = 79.2, *p*-value = 2e^-16^, n = 20) followed by Tukey’s test, significant differences between isolates are represented by letters.

Two weeks after injection, the bacterial-inoculated potato plants showed black rotting on the lower part of the stem (**Figure 3A**). Bacteria were isolated from the stem lesions and identified using *rpoS* and *gapA* primers as identical to the BY21311 used for inoculation. Therefore, *P. polonicum* isolate BY21311 fulfills Koch’s postulates for potato blackleg. Maceration assays were also performed to evaluate the virulence of isolate BY21311 on potato tubers by comparing the macerated tissue weights. Seventy-two hours after inoculation, all *Pectobacterium* species caused soft rot on potato tubers, whereas the buffer (10 mM MgSO_4_) control group exhibited only a very small amount of tissue debris in the cavity (0.03 ± 0.09 g/tuber). Significant variations in maceration ability between *Pectobacterium* species were observed (the *p*-value of the *F*-test [*F* = 79.2] was 2e^-16^) (**Figure 3B**). *P. atrosepticum* and *P. parvum* exhibited the highest and lowest virulence level on tubers, respectively. *P. atrosepticum* isolate FN20412 caused the most significant amount (4.37 ± 0.78 g/tuber) of maceration on potato tubers; followed by *P. versatile* isolate FN20111 and *P. brasiliense* isolate FR19412, which caused 3.26 ± 0.80 g/tuber and 2.73 ± 0.57 g/tuber, respectively. *P. parvum* isolate FN20211 (1.67 ± 0.58 g/tuber), and *P. polonicum* isolate BY21311 (1.23 ± 0.53 g/tuber) caused a relatively low amount of maceration, and the virulence of *P. polonicum* isolate BY21311 was slightly less than *P. parvum* on tubers, although the adjusted *p*-value (0.14) did not reach significance. However, the low virulence exhibited by *P. polonicum* on potato tuber in this study does not agree with previous study in that *P. polonicum* and *P. atrosepticum* showed equal virulence on potato slices (Waleron et al., 2019).

### HR in N. benthamiana

**Figure 4:**
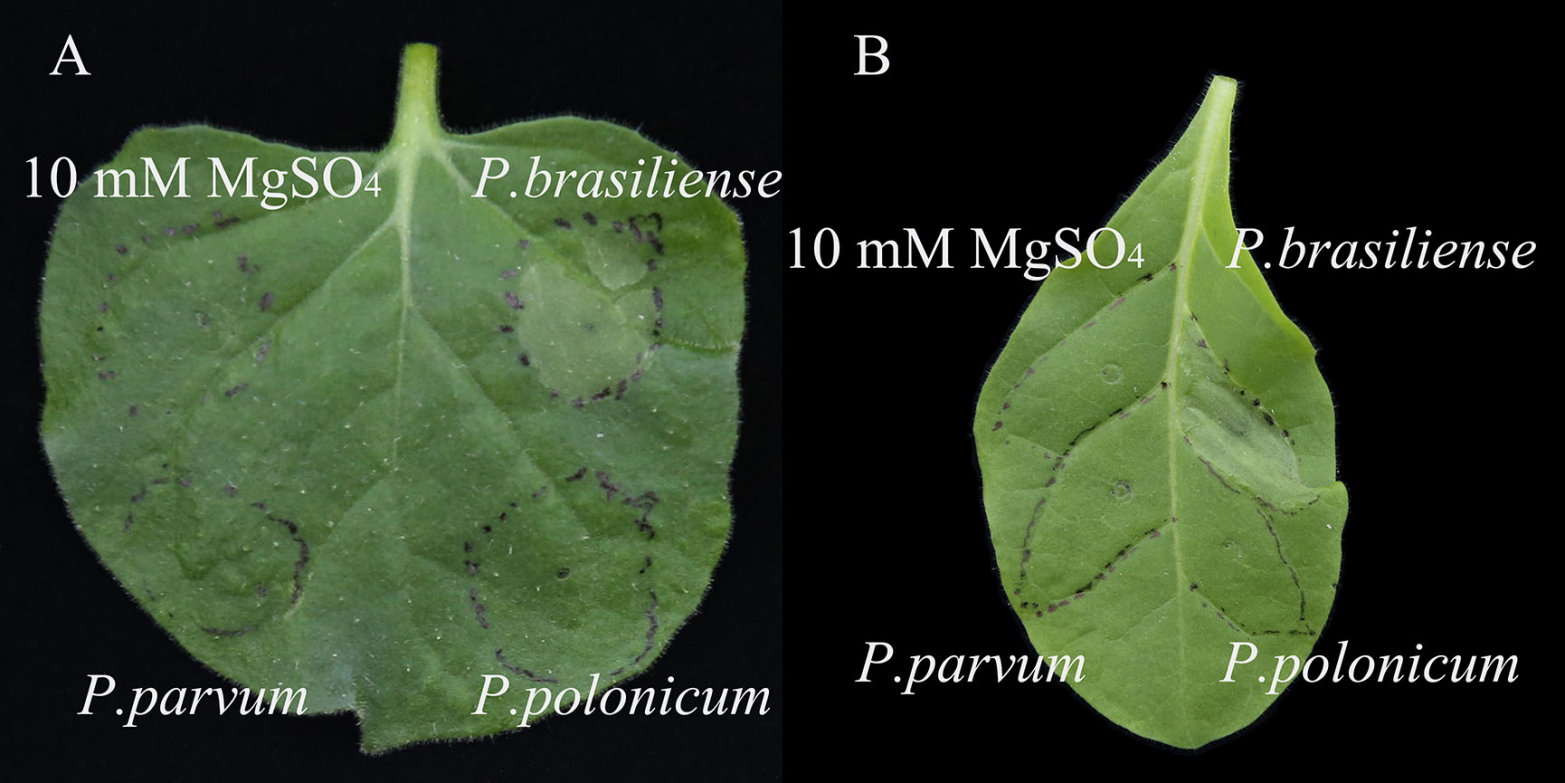
Hypersensitive response in **(A)** *Nicotiana benthamiana* and **(B)** *N. tabacum* leaves infiltrated with the *Pectobacterium* species. *P. brasiliense* isolate FR19412 and *P. parvum* isolate FN20211 were used as positive and negative controls, respectively. 10 mM MgSO_4_ was used as a buffer control. Leaves were detached and photographed 48 hours after infiltration. Similar results were obtained in two independent experiments.

**Figure 5:**
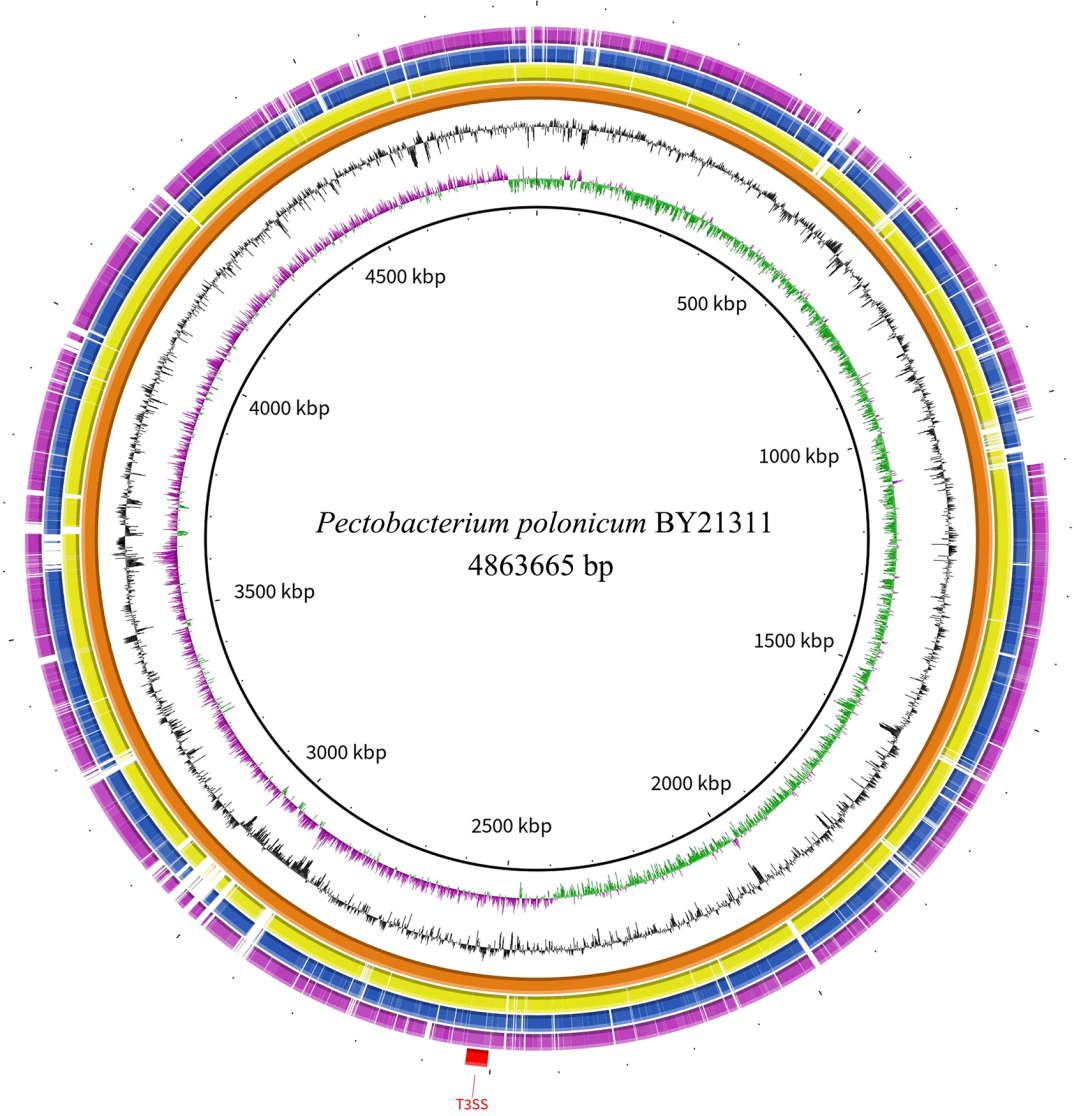
Circular representation of genome sequences of the *Pectobacterium polonicum* isolate BY21311, *P. polonicum* type strain DPMP315^T^, *P. punjabense* type strain SS95^T^, and *P. brasiliense* type strain LMG21371^T^. The rings represent (from inner to outer) GC skew, GC%, the whole genome sequence of isolate BY21311, corresponding homologous regions in *P. polonicum* DPMP315^T^, *P. punjabense* SS95^T^ and *P. brasiliense* LMG21371^T^, and the genome region encoding type III secretion system (T3SS). Genome comparison was created using BRIG v0.95.

The ability of isolate BY21311 to cause an HR was tested in non-host tobacco plants, *N. benthamiana* and *N. tabacum*. After 24 h, water-soaking lesions only appeared on tobacco leaves at sites infiltrated with *P. brasiliense* isolate FR19412 (**Figures 4A, B**). *P. parvum* isolate FN20211 and *P. polonicum* isolate BY21311 did not able to elicit HR in tobacco leaves. The genome of *P. polonicum* isolate BY21311 harbors gene clusters (2,504,628.2,537,200) encoding the T3SS apparatus which was also found in the type strain DPMP315^T^ and sister species *P. punjabense* SS95^T^ (**Figure 5**).

### Host range determination

**Figure 6:**
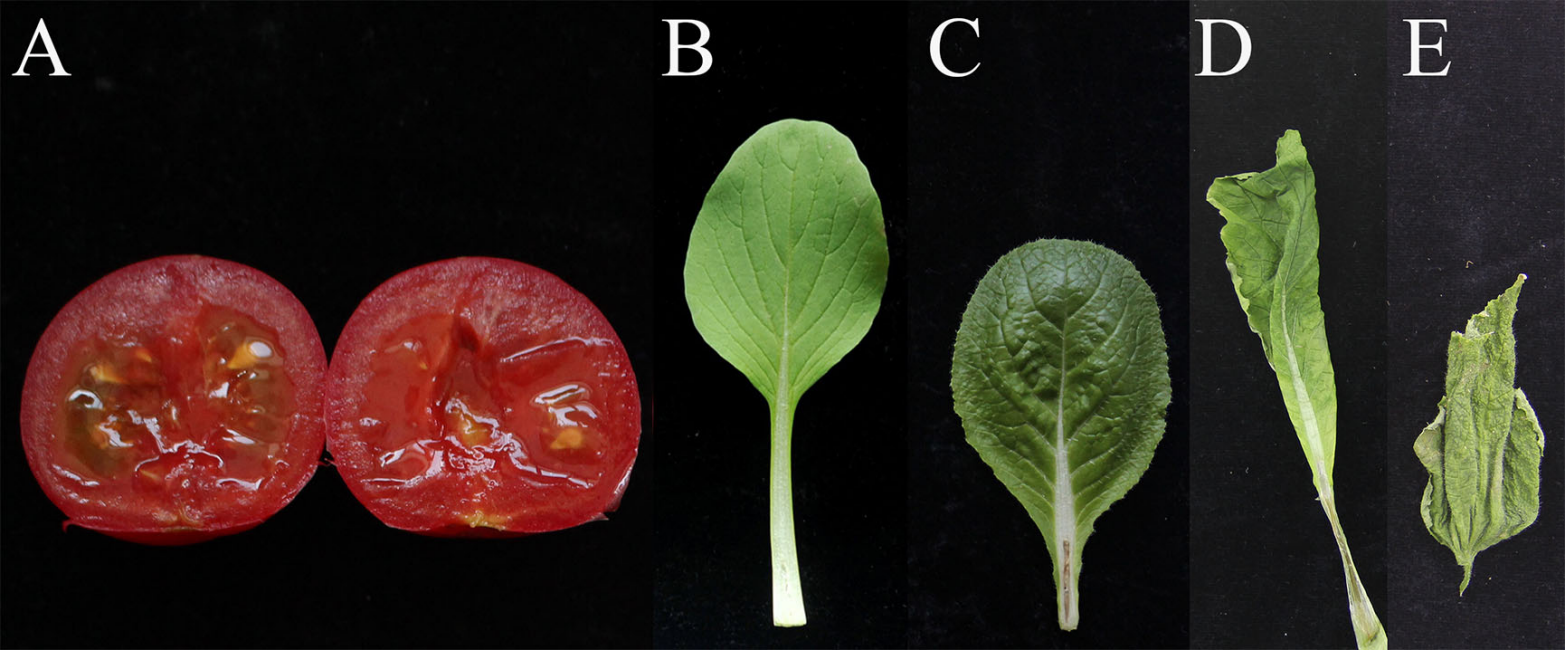
P*. polonicum* isolate BY21311 host range determined. The stems of vegetables were detached and photographed 7days after infiltration. Washed and surface sterilized tomato were inoculated with isolate BY21311 and cut open at 3 days after inoculation. **(A)** tomatoes (*Solanum lycopersicum* L.) **(B)** *Brassica chinensis* L. **(C)** baby chinese cabbage, **(D)** baby bok choy (*Brassica campestris* L.ssp*.chinensis* Makino var.*communis* Tsen et Lee), **(E)** *Cucumis sativus* L.

After inoculation of BY21311 for 3 days or 7 days by injection, all vegetables showed disease of soft rot, and *Brassica chinensis* L. had the mildest symptoms (**Figure 6**). The results suggest that these vegetables may be potential hosts for isolate BY21311.

## Discussion

In northern China’s Hebei Province, stem rot diseases were observed in potato fields in Zhangjiakou, Tangshan and Baoding in 2021, Five *Pectobacterium* species were identified in diseased plants by MLSA, including *P. carotovorum*, *P. atrosepticum*, *P. brasiliense*, *P. parmentieri* and *P. polonicum* (**Table 1**; **Supplementary Table S1**; **Supplementary Figure 1**). Four of the identified *Pectobacterium* species have been recognized as pathogens in potato plants; however, *P. polonicum* was first detected in groundwater in Poland (Waleron et al., 2019), appearing to be a water-associated bacterium. The experiment of artificial inoculation of potato tubers showed that *P. atrosepticum* and *P. parvum* exhibited the highest and lowest virulence level on tubers, respectively. This result agrees with previous studies (Pasanen, 2020; Cigna et al., 2021). *P. atrosepticum* isolate FN20412 caused the most significant amount of maceration on potato tubers, followed by *P. versatile* isolate FN20111 and *P. brasiliense* isolate FR19412. *P. parvum* isolate FN20211, and *P. polonicum* isolate BY21311 caused relatively low amounts of maceration.

The low virulence exhibited by *P. polonicum* on potato tubers in this study does not agree with a previous study in which *P. polonicum* and *P. atrosepticum* showed equal virulence on potato slices (Waleron et al., 2019). Artificial inoculation showed that *P. polonicum* could macerated potato tubers, calla lily, and chicory leaves (Waleron et al., 2019), this study show that *P. polonicum* can also macerated baby bok choy, baby Chinese cabbage, *Brassica chinensis* L., *Cucumis sativus* L. and tomatoes, suggesting that it might be a potential plant pathogen; however, there have been no *P. polonicum*-related plant diseases reported to date, and its pathogenicity in plants was yet to be determined. In the present study, *P. polonicum* was found in potato plants in the Baoding region in China (**Supplementary Table S1**). The blackleg incidence in that field was approximately 40%, and *P. carotovorum* was found in the same field; therefore, the disease was most likely caused by a mixture of *Pectobacterium* species. The taxonomic position of isolate BY21311 was further confirmed using WGS and genome-based analysis, and the results revealed that the isolate belonged to *P. polonicum* (**Figures 1**, **2**). In conditions of artificial inoculation in a greenhouse fulfilled Koch’s postulates for potato blackleg (**Figure 3**). For the first time, *P. polonicum* has been demonstrated to be an opportunistic pathogen of potato blackleg in the field. It remains unknown whether *P. polonicum* is restricted to a small, local geographic area in China; nevertheless, this new emerging pathogen poses a potential threat to potato production.

Some members of the genus *Pectobacterium* has been associated with plant diseases, and some have a broad host range. In contrast, four *Pectobacterium* species have been identified only from non-host environments such as water, including *P. fontis* (Oulghazi et al., 2019), *P. aquaticum* (Pedron et al., 2019), *P. polonicum* (Waleron et al., 2019), and *P. quasiaquaticum* (Moussa et al., 2021). *P. fontis* exhibited less maceration ability in potato tubers than the plant-associated *Pectobacterium* species (Oulghazi et al., 2019), and similar pathogenic traits were also observed in *P. polonicum* BY21311. However, reduced virulence in tubers is not a universal trait in the water-related species; at least, it does not apply to *P. polonicum* DPMP315^T^ and others (Waleron et al., 2019). *P. polonicum* appears to have significant variations in gene content between the two genomes that facilitate rapid adaptation to changing environments by acquiring of genetic material. Thus, it is possible that BY21311 and DPMP315^T^ may represent two distinct clades within *P. polonicum* because they varied in many aspects on the genome comparison level, including LPS and LPS O-antigen biosynthesis, substrate translocation, T4SS, and T6SS, among others. Among our predicted effectors, AvrE-famely type III effector is widespread among type III-dependent phytobacteria and is the most critical virulence factor in plant pathogens (Bogdanove et al., 1998a; Bogdanove et al., 1998b). They inhibit salicylic acid-mediated plant defense, interfere with blister transport, promote plant bacterial growth, and elicit plant cell death (Degrave et al., 2015). PilN is an important type IV fimbrial assembly family protein, and its function is mainly related to the biosynthesis of Pili (Tammam et al., 2011; Kim and Komano, 1997). Wang constructed *PilN* gene mutants and found that the mutants’ pathogenicity, wandering ability and extracellular cellulolytic activity were reduced, and tobacco anaphylaxis was lost. The results showed that *pilN* gene related to pili biosynthesis plays an important role in regulating pathogenicity (Wang et al., 2013).

The closest species to *P. polonicum* is *P. punjabense* (Sarfraz et al., 2018), which can also cause potato blackleg; the species displayed similar physiological and biochemical characteristics (**Table 2**). The HR experiment in non-host plants showed that *P. brasiliense* elicited HR in *N. benthamiana* and *N. tabacum* leaves, but *P. parvum* did not. This result agrees with previous studies (Kim et al., 2009; Pasanen et al., 2020; Maphosa and Moleleki, 2021). Interestingly, *P. polonicum* BY21311 and *P. parvum* share common phenotypic characteristics of low virulence on potato tubers (**Figure 3**) and absence of HR on tobacco leaves (**Figure 4**). *Pectobacterium* species do not require the T3SS for the pathogenicity, but HR assay has been widely used as an indicator for a functional T3SS (Kim et al., 2009). *P. parvum* genome encodes a *Salmonella* SP-1-like T3SS, most likely involved in direct interactions with insect vectors but not with host plants (Pasanen et al., 2020). The genomes of *P. polonicum* DPMP315^T^ and BY21311 harbor gene clusters encoding a T3SS also found in the genome of *P. punjabense* (**Figure 5**); however, isolate BY21311 failed to elicit HR in tobacco, suggesting its T3SS might not function. All previously reported *P. parvum* isolates were associated only with aerial stem rot in potato plants (Pasanen et al., 2013; Pasanen et al., 2020; Wang et al., 2022a). The low virulence on tubers and association with stem rot suggests potential convergent evolution in *P. parvum* and *P. polonicum* that may have developed tissue specificity.

Blackleg and aerial stem rot of potatoes caused by SRP have become more frequent in China in recent years (Zhao et al., 2018; Cao et al., 2021; Han et al., 2022; Handique et al., 2022a; Wang et al., 2022a; Handique et al., 2022b). Potato stem rot caused by *P. versatile* (Han et al., 2022) and *P. parvum* (Wang et al., 2022a) was reported in the Fengning region in the summer of 2020; therefore, seven *Pectobacterium* species have been identified in potato plants in Hebei Province to date. MLSA demonstrated that a greater diversity of *Pectobacterium* infects potatoes at the province scale than previously reported: *P. carotovorum*, *P. atrosepticum*, *P. brasiliense*, *P. parmentieri*, *P. polaris* and *P. punjabense* in Guangdong, Inner Mongolia and Sichuan (Zhang et al., 2012; She et al., 2013; Zhao et al., 2018; Jiang et al., 2019; Cao et al., 2021; Handique et al., 2022a; Handique et al., 2022b). However, the *P. carotovorum* complex has been revised several times, and it is possible that some earlier isolated *P. carotovorum* strains might have been misclassified. On the other hand, the international exchange of crop germplasm and global seed trade could facilitate the spread of seed-borne pathogens. It is possible that some species of SRP were introduced along with the importation of seed potatoes because China’s potato industry heavily relies on imported commercial varieties which are mainly imported from the Netherlands, the United States, and Canada (Wang et al., 2019). For example, Helan 15, the most widely grown variety in China over the past two decades, actually is Favorita (HZPC, the Netherlands), which was first introduced into mainland China in the 1980s. Although it is uncertain whether any of the SRP isolates entered China *via* seed importation, nevertheless, susceptible cultivars acted as one of the primary causes of disease prevalence in this case. Production of Xisen 6 and Huangxin 226 has expanded rapidly in recent years in China due to the consumer preference. Unfortunately, these cultivars are highly susceptible to *Pectobacterium* species, which have been reported in Guangdong (Jiang et al., 2019), Hebei (Han et al., 2022; Wang et al., 2022a), and Inner Mongolia (Cao et al., 2021).

## Data availability statement

The datasets presented in this study can be found in online repositories. The names of the repository/repositories and accession number(s) can be found in the article/**Supplementary Material**.

## Author contributions

JHW, WXH, and JHZ conceived and designed the experiments and analyzed the data. WXH, YP, JXQ, SQZ and ZHY performed the experiments. JHW wrote the paper. MP has revised this paper. All the authors reviewed the manuscript.
