# Supplementary material for: Identification and characterization of opportunistic pathogen Pectobacterium polonicum causing potato blackleg in China
Source: Front Plant Sci. 2023 Mar 3;14:1097741. doi: 10.3389/fpls.2023.1097741 (PMC10020715; doi:10.3389/fpls.2023.1097741)
Supplement: Supplementary file 1 [file DataSheet_1.docx]

Supplementary Material

# Supplementary Figures and Tables

## Supplementary Tables

**Supplementary Table S1.** Pectinolytic isolates identified by MLSA in this study.

| **Isolate ID** | **Sample collection date** | **Location** | **Disease incidence**  **in field** | **Disease symptom^a^** | **Species assignation based on MLSA^b^** | **16S accession number (GenBank)** | **Best hit in NCBI 16S rRNA database**  **(%identity)** | **MLSA accession number (GenBank)** |
| --- | --- | --- | --- | --- | --- | --- | --- | --- |
| YT21111 | 16-05-2021 | Yutian, Tangshan | 25% | BL | Pat | OL958693  (1357bp) | NR_118295.1 *P. atrosepticum* CFBP1526 100% | OM044527-OM044532 |
| YT21121 | 16-05-2021 | Yutian, Tangshan |  | BL | Pat | OL958694  (1357bp) | NR_118295.1 *P. atrosepticum* CFBP1526 100% | OM044533-OM044538 |
| YT21211 | 16-05-2021 | Yutian, Tangshan |  | BL | Ppm | OL958695  (1314bp) | NR_153752.1 *P. parmentieri* RNS08-42-1A 99.85% | OM044539-OM044544 |
| YT21222 | 16-05-2021 | Yutian, Tangshan |  | BL | Ppm | OL958696  (1314bp) | NR_153752.1 *P. parmentieri* RNS 08-42-1A 99.85% | OM044545-OM044550 |
| BY21311 | 26-05-2021 | Boye, Baoding | 40% | BL | Ppl | OL958686  (1389bp) | NR_118294.1 *P. wasabiae* CFBP3304 98.85% | OM044551-OM044556 |
| BY21312 | 26-05-2021 | Boye, Baoding |  | BL | Ppl | OL958687  (1389bp) | NR_118294.1 *P. wasabiae* CFBP3304 98.85% | OM044557-OM044562 |
| BY21121 | 26-05-2021 | Boye, Baoding |  | BL | Pca | OL958684  (1320bp) | NR_118227.1 *P. carotovorum* CFBP2046 99.02% | OM044563-OM044568 |
| BY21221 | 26-05-2021 | Boye, Baoding |  | BL | Pca | OL958685  (1349bp) | NR_118227.1 *P. carotovorum* CFBP2046 99.02% | OM044569-OM044574 |
| ZB21311 | 19-07-2021 | Zhangbei, Zhangjiakou | 30% | ASR | Pbr | OL958697  (1362bp) | NR_159925.1 *P. aroidearum* SCRI109 99.85% | OM044575-OM044580 |
| ZB21312 | 19-07-2021 | Zhangbei, Zhangjiakou |  | ASR | Pbr | OL958698  (1362bp) | NR_159925.1 *P. aroidearum* SCRI109 99.85% | OM044581-OM044586 |

^a^ ASR: aerial stem rot; BL: blackleg

^b^ Pat: *Pectobacterium atrosepticum*; Ppm: *P.* *parmentieri*; Ppl: *P. polonicum*; Pca: *P. carotovorum*; Pbr: *P. brasiliense*

**Supplementary Table S2.** RefSeq genome datasets used in this study.

| **Organism Name** | **Strain^a^** | **Size (Mb)** | **GC%** | **RefSeq Assembly** |
| --- | --- | --- | --- | --- |
| *Pectobacterium fontis* | M022^T^ | 4.15 | 51.20 | GCF_000803215.1 |
| *Pectobacterium carotovorum* | DSM 30168^T^ | 4.76 | 51.90 | GCF_900129615.1 |
| *Pectobacterium carotovorum* | CFBP7347 | 4.69 | 52.00 | GCF_013449395.1 |
| *Pectobacterium carotovorum* | ICMP 5702 | 4.77 | 51.90 | GCF_001039055.1 |
| *Pectobacterium atrosepticum* | CFBP1526^T^ | 5.04 | 51.06 | GCF_019056595.1 |
| *Pectobacterium atrosepticum* | NCPPB 549^T^ | 5.10 | 50.90 | GCF_000749905.1 |
| *Pectobacterium brasiliense* | LMG 21371^T^ | 4.83 | 52.10 | GCF_000754695.1 |
| *Pectobacterium brasiliense* | BC1 | 4.92 | 51.80 | GCF_001932635.1 |
| *Pectobacterium brasiliense* | SX309 | 4.97 | 52.20 | GCF_002068115.1 |
| *Pectobacterium parmentieri* | RNS 08-42-1A^T^ | 5.03 | 50.40 | GCF_001742145.1 |
| *Pectobacterium parmentieri* | SCC3193 | 5.16 | 50.40 | GCF_000260925.1 |
| *Pectobacterium wasabiae* | CFBP 3304^T^ | 5.04 | 50.60 | GCF_001742185.1 |
| *Pectobacterium wasabiae* | NCPPB 3701 | 5.01 | 50.50 | GCF_000749865.1 |
| *Pectobacterium aroidearum* | L6 | 5.00 | 51.80 | GCF_015689195.1 |
| *Pectobacterium aroidearum* | LJ2 | 4.86 | 51.90 | GCF_020181375.1 |
| *Pectobacterium actinidiae* | KKH3^T^ | 4.92 | 51.50 | GCF_000803315.1 |
| *Pectobacterium actinidiae* | ICMP 19972 | 4.89 | 51.50 | GCF_001984525.1 |
| *Pectobacterium odoriferum* | NCPPB 3839^T^ | 5.07 | 51.60 | GCF_000754765.1 |
| *Pectobacterium odoriferum* | JK2.1 | 5.10 | 51.53 | GCF_009931295.1 |
| *Pectobacterium versatile* | CFBP6051^T^ | 4.95 | 51.90 | GCF_004296685.1 |
| *Pectobacterium versatile* | SCC1 | 4.98 | 51.90 | GCF_003571485.1 |
| *Pectobacterium zantedeschiae* | 9M^T^ | 5.06 | 50.72 | GCF_004137795.1 |
| *Pectobacterium zantedeschiae* | PC2 | 5.09 | 50.40 | GCF_004137785.1 |
| *Pectobacterium punjabense* | SS95^T^ | 4.79 | 50.70 | GCF_012427845.1 |
| *Pectobacterium punjabense* | IPO:3715 SV1490b | 4.74 | 50.90 | GCF_016950155.1 |
| *Pectobacterium peruviense* | IFB5232^T^ | 4.80 | 51.10 | GCF_002847345.1 |
| *Pectobacterium peruviense* | UGC32 | 4.80 | 51.10 | GCF_001039065.1 |
| *Pectobacterium betavasculorum* | NCPPB 2795^T^ | 4.69 | 51.20 | GCF_000749845.1 |
| *Pectobacterium betavasculorum* | NCPPB 2793 | 4.68 | 51.00 | GCF_000749925.1 |
| *Pectobacterium polaris* | NIBIO1006^T^ | 4.83 | 52.00 | GCF_002307355.1 |
| *Pectobacterium polaris* | NIBIO1392 | 5.01 | 52.00 | GCF_002288545.1 |
| *Pectobacterium quasiaquaticum* | A477-S1-J17 | 4.40 | 51.70 | GCF_014946775.1 |
| *Pectobacterium quasiaquaticum* | A398-S21-F17^T^ | 4.25 | 51.70 | GCF_014946825.1 |
| *Pectobacterium parvum* | s0421^T^ | 4.70 | 51.00 | GCF_900195285.2 |
| *Pectobacterium parvum* | s0416 | 4.61 | 51.00 | GCF_900195295.2 |
| *Pectobacterium aquaticum* | A212-S19-A16^T^ | 4.35 | 51.20 | GCF_003382565.2 |
| *Pectobacterium aquaticum* | A101-S19-F16 | 4.27 | 51.50 | GCF_003382625.2 |
| *Pectobacterium polonicum* | DPMP315^T^ | 4.84 | 51.28 | GCF_005497185.1 |
| *Dickeya solani* | IPO 2222^T^ | 4.92 | 56.20 | GCF_001644705.1 |
| *Dickeya dadantii* | DSM 18020^T^ | 5.00 | 56.40 | GCF_003049785.1 |

^a^ The superscript ‘T’ indicates type strain.

**Supplementary Table S3.** General genome features of *Pectobacterium polonicum* isolates BY21311 and type strain DPMP315^T^, and *P. punjabense* type strain SS95^T^.

|  | **Accession number** | **Length (bp)** | **GC%** | **CDSs**  **(protein)** | **rRNAs** | **tRNAs** | **BUSCO (Enterobacterales)** |
| --- | --- | --- | --- | --- | --- | --- | --- |
| BY21311 | CP090065.1 | 4,863,665 | 51.08 | 4,178 | 22 | 77 | 99.32%  (437/440) |
| *P. polonicum* DPMP315^T^ | NZ_RJTN00000000.1 | 4,836,128 | 51.30 | 4,314 | 18 | 72 | 99.32%  (437/440) |
| *P. punjabense* SS95^T^ | CP038498.1 | 4,793,778 | 50.70 | 4,183 | 22 | 77 | 99.77%  (439/440) |

**Supplementary Table S4.** genome comparison analysis of *Pectobacterium polonicum* isolates DPMP315^T^ and BY21311.

**Supplementary Table S5.** Sequences of PCR primers used in this study.

| **Target gene** | **Primer name** | **Sequences (5’-3’)** | **Reference** |
| --- | --- | --- | --- |
| 16s rDNA | 27F | AGAGTTTGATCCTGGCTCAG | Weisburg et al. 1991 |
|  | *1492R* | GGTTACCTTGTTACGACTT |  |
| *acnA* | *acnA_F* | CMAGRGTRTTRATGCARGAYTTTAC | Ma et al. 2007 |
|  | *acnA_R* | GATCATGGTGGTRTGSGARTCVGT |  |
| *gapA* | *gapA_F* | ATCTTCCTGACCGACGAAACTGC | Ma et al. 2007 |
|  | *gapA_R* | ACGTCATCTTCGGTGTAACCCAG |  |
| *icdA* | *icdA_F* | GGTGGTATCCGTTCTCTGAACG | Ma et al. 2007 |
|  | *icdA_R* | TAGTCGCCGTTCAGGTTCATACA |  |
| *mdh* | *mdh_F* | CCCAGCTTCCTTCAGGTTCAGA | Ma et al. 2007 |
|  | *mdh_R* | CTGCATTCTGAATACGTTTGGTCA |  |
| *proA* | *proA_F* | CGGYAATGCGGTGATTCTGCG | Ma et al. 2007 |
|  | *proA_R* | GGGTACTGACCGCCACTTC |  |
| *rpoS* | *rpoS_F* | ATGAGCCAAAGTACGCTGAA | Waleron et al. 2008 |
|  | *rpoS_R* | ACCTGAATCTGACGAACACG |  |

## Supplementary Figures


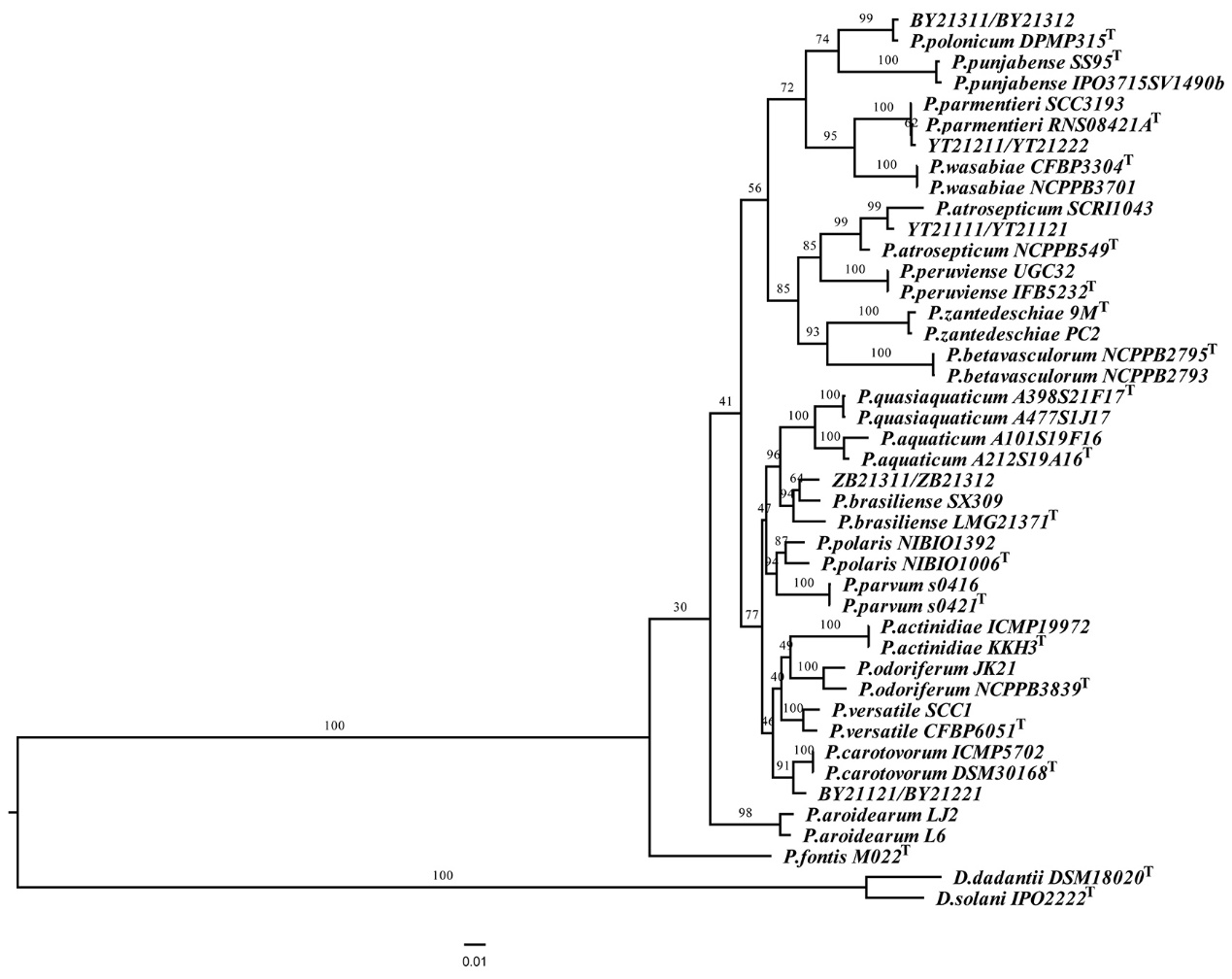


**Supplementary Figure 1.** Phylogenetic tree showing the phylogenetic relationship between *Pectobacterium* isolates BY21311 and BY21312 to their closely related *Pectobacterium* species, based on the concatenated sequences of six loci, *acnA*, *gapA*, *icdA*, *mdh*, *proA*, and *rpoS* (3228 bp). *Dickeya solani* and *D. dadantii* were selected as outgroups. The maximum likelihood method was used to reconstruct the phylogenetic tree was constructed using RAxML v8.2.12 with the ‘GTRGAMMAI’ setting. The superscript ‘T’ in the tree indicates type strain. The branch lengths indicate the evolutionary distance as the number of base substitutions per site. The numbers shown next to the branches indicate the percentage of bootstrap support values (1000 replicates).
